# A hybrid Daubechies wavelet collocation approach for a fractional-order SIR epidemic model with delay effects

**DOI:** 10.1038/s41598-025-32483-z

**Published:** 2025-12-20

**Authors:** Nimai Sarkar, Mausumi Sen

**Affiliations:** 1 School of Advanced Sciences, VIT-AP University, Inavolu, Amaravati, 522237 Andhra Pradesh India; 2 Department of Mathematics, National Institute of Technology Silchar, Silchar, 788010 Assam India

**Keywords:** Fractional-order SIR model, Time delay, Daubechies wavelets, Hybrid collocation method, Caputo derivative, Epidemic modeling, Engineering, Mathematics and computing, Physics

## Abstract

This paper studies the transmission dynamics of influenza by using a fractional SIR (Susceptible-Infected-Removed) epidemic model with discrete delay to describe the short-term dynamics. The model includes history-dependent effects through Caputo fractional derivative and maturity delays, which are biologically motivated as the incubation periods or delayed immune responses. In this paper, we will solve this model by introducing a hybrid collocation method with the Daubechies wavelet basis that can be used to efficiently take into account the fractional-order system and the delay system. The reliability and efficiency of the presented algorithm are investigated by means of comparison with some well-known numerical methods, such as the classical Runge-Kutta method (RK4), the Rational Polynomial Spectral Method of order 7 (RPSM7), the Generalized Wavelet Collocation Method (GWCM), and the Genocchi wavelet method. Numerical simulations demonstrate. Our Daubechies wavelet-based method is reported to converge more. Stably and better track both memory and delay effects in the context of the numerical simulations. Nonetheless, the technique presumes fixed parameters (specifically transmission rates), simplifying the situation of multiple unknown parameter values, which are often encountered. Such heterogeneity, particularly in transmission rates, is likely to impact the model’s predictions and should be accounted to have a better and realistic epidemic model. In addition, the method’s efficiency may increase in the case of systems on a large scale or real-time simulations. However, it offers a higher approximation accuracy with lower computational overhead when compared to the known methods.

## Introduction

Epidemiology is a key branch of biology that focuses on studying the occurrence and distribution of health-related issues among specific groups or regions^1^. In this context, an epidemic refers to the rapid spread of a viral disease affecting a large population over a short period. Such rapid transmission tends to happen when host immunity plummets starkly — via, say, the arrival of a fresh pathogen or, for that matter, the return of an old one. Epidemiology can underpin public health policies with statistics that feed into the procedure for planning and controlling interventions to address population diseases. It is essential for the study and explanation of disease onset, or decline, to the community, and for the comparison of risk factors or other exposures at the individual level, as well as for the monitoring of the evolution of disease^2^.

Mathematical models offer an organized way to comprehend real epidemics. Researchers can use mathematical equations to translate the biological processes, simulate the disease dynamics, predict invasion, and assess control strategies by translating biological processes into mathematical equations. Many research works have utilized ordinary differential equations (ODEs) to model and analyze infectious diseases^3–5^. One of the most critical and influential models is the SIR model by Kermack and McKendrick, which can be considered its forerunner. These models have then gradually expanded to make it possible to consider more realistic aspects, like memory effect and time delay.

Fractional calculus (FC) provides powerful tools for modeling hereditary and memory effects^6–10^, which are often present in real-world disease spread. FC allows for derivatives and integrals of non-integer order, enabling a more accurate representation of dynamics over time^11,12^. Modified operators, such as the Caputo, Riemann–Liouville, and Atangana–Baleanu derivatives, are frequently employed to capture these nonlocal effects^13,14^. Among these, the Caputo derivative is especially useful in initial value problems due to its compatibility with classical initial conditions. It enables smooth and continuous solution behavior, which aligns well with the nature of disease progression.

Fractional-order models using the Caputo operator have gained popularity in epidemiology. For example, models using Atangana–Baleanu operators have been used to study computer virus outbreaks^4^ and the Zika virus^5^. Such models often incorporate time delays^15,16^ to represent incubation periods, delayed immune responses, or waning immunity. Delay terms bring these models closer to biological reality and can introduce rich dynamical behaviors such as oscillations and multi-peak outbreaks.

Wavelet-based numerical methods have also emerged as highly efficient techniques for solving fractional differential equations. Wavelets provide compact support, orthogonality, and multiresolution analysis, which are ideal properties for capturing the localized behavior of epidemic outbreaks. Various wavelet techniques—such as Fibonacci, Chebyshev, Bernoulli, and Haar—have been applied in the literature to simulate complex systems^17–20^.

A notable development in this area is the Genocchi Wavelet Collocation Method (GWCM)^21–24^, which has been effectively used to solve SIR models involving fractional-order dynamics and delay effects^25,26^. The Genocchi wavelets offer compact representation, reduced computational cost, and better handling of discontinuities compared to classical polynomial bases. Their operational matrices allow fractional derivatives to be approximated efficiently, converting the model into an algebraic system that is straightforward to solve.

While GWCM has shown excellent results, our approach is motivated by the potential of Daubechies wavelets, which offer greater smoothness and higher-order vanishing moments. This study presents a hybridized collocation method using Daubechies wavelets to solve a fractional-order SIR model with a discrete delay. Our process integrates the strengths of both spectral collocation and wavelet theory. The proposed Daubechies-based collocation method accurately captures memory and delay effects while reducing memory usage and computational load.

Unlike traditional methods^27–35^, the proposed scheme does not require storing the entire history of the system. The compact support and smooth basis of Daubechies wavelets allow us to resolve sharp gradients and delayed responses with fewer computational resources. Furthermore, we ensure stability and spectral accuracy by hybridizing collocation with wavelet decomposition. Comparative analysis shows that our method outperforms RK4, RPSM7, GWCM, and other standard techniques, especially in long-term simulations with significant memory effects.

The structure of this paper is as follows. Section 2 reviews basic definitions of fractional calculus and wavelets. Section 3 introduces the influenza epidemic model with delay. Section 4 details the construction of the hybridized Daubechies wavelet collocation scheme (HDWCS). Section 5 presents numerical simulations and comparative results. Finally, Section 6 summarizes the findings and future directions.

## Preliminaries

This section presents essential mathematical tools and definitions that are used in formulating and solving the proposed fractional-order SIR model with delay. We begin with definitions from fractional calculus, followed by properties of the Caputo derivative and an overview of Daubechies wavelets and their operational matrices.

### Definition 1

(Riemann–Liouville Fractional Integral^11^) Let $f \in L^1[a,b]$ and $\alpha> 0$. The left-sided Riemann–Liouville fractional integral is defined by

$$\begin{aligned} (I_a^\alpha f)(t) = \frac{1}{\Gamma (\alpha )} \int _a^t (t-\tau )^{\alpha -1} f(\tau ) \, d\tau . \end{aligned}$$

### Definition 2

(Caputo Fractional Derivative^13^) Let $f \in C^n[a,b]$, $n = \lceil \alpha \rceil$. The Caputo fractional derivative is defined by

$$\begin{aligned} (^C D_a^\alpha f)(t) = \frac{1}{\Gamma (n - \alpha )} \int _a^t (t - \tau )^{n - \alpha - 1} f^{(n)}(\tau ) \, d\tau . \end{aligned}$$

### Definition 3

(Atangana–Baleanu Fractional Derivative^14^) The Atangana–Baleanu Caputo derivative is given by

$$\begin{aligned} ^{ABC} D_a^\alpha f(t) = \frac{B(\alpha )}{1 - \alpha } \int _a^t f^\prime(\tau ) E_\alpha \left( -\frac{\alpha }{1 - \alpha }(t - \tau )^\alpha \right) \, d\tau , \end{aligned}$$

where $E_\alpha (\cdot )$ is the Mittag-Leffler function.

### Proposition 2.1

(Linearity^12^) For scalars $a,b \in \mathbb {R}$ and functions *f*, *g* such that the fractional derivative exists:

$$\begin{aligned} D^\alpha (a f + b g)(t) = a D^\alpha f(t) + b D^\alpha g(t). \end{aligned}$$

### Proposition 2.2

(Semigroup Property^38^) If $\alpha ,\beta> 0$ and *f* is sufficiently smooth, then

$$\begin{aligned} D^\alpha D^\beta f(t) = D^{\alpha +\beta } f(t). \end{aligned}$$

### Proposition 2.3

(Identity Property^11^)

$$\begin{aligned} D^\alpha I^\alpha f(t) = f(t), \quad I^\alpha D^\alpha f(t) = f(t) \text { under suitable regularity conditions}. \end{aligned}$$

### Definition 4

(Daubechies Wavelet Basis^39^) Daubechies wavelets $\psi (t)$ are orthonormal functions with compact support and *N* vanishing moments:

$$\begin{aligned} \int t^k \psi (t) \, dt = 0, \quad \text {for } k = 0, 1, \dots , N - 1. \end{aligned}$$

They form a basis for $L^2(\mathbb {R})$ via scaling and translation:

$$\begin{aligned} \psi _{j,k}(t) = 2^{j/2} \psi (2^j t - k). \end{aligned}$$

### Proposition 2.4

(Multi-resolution Analysis^40^) Let $V_j$ be the subspace generated by scaling functions. Then Daubechies wavelets satisfy:

$$\begin{aligned} \dots \subset V_{-1} \subset V_0 \subset V_1 \subset \dots , \quad L^2(\mathbb {R}) = \overline{\bigcup _j V_j}. \end{aligned}$$

A function $f(t) \in L^2(\mathbb {R})$ can be approximated as:

$$\begin{aligned} f(t) \approx \sum _{k=0}^{2^J - 1} c_k \phi _{J,k}(t) + \sum _{j=J}^{J + M} \sum _{k=0}^{2^j - 1} d_{j,k} \psi _{j,k}(t), \end{aligned}$$

where $\phi _{J,k}(t)$ and $\psi _{j,k}(t)$ are scaling and wavelet functions, respectively.

To solve fractional differential equations, we write:

$$\begin{aligned} f(t) \approx \sum _{i=1}^n c_i \psi _i(t), \quad D^\gamma f(t) \approx \sum _{i=1}^n c_i D^\gamma \psi _i(t). \end{aligned}$$

### Definition 5

(Operational Matrix^36^) The matrix $\textbf{D}_\gamma$ with entries

$$\begin{aligned} [\textbf{D}_\gamma ]_{ij} = \langle D^\gamma \psi _j(t), \psi _i(t) \rangle \end{aligned}$$

approximates the fractional derivative as

$$\begin{aligned} D^\gamma f(t) \approx \textbf{D}_\gamma C, \quad C = [c_1, \dots , c_n]^T. \end{aligned}$$

### Definition 6

(Caputo Derivative of Basis^37^) The Caputo derivative of wavelet $\psi _j(t)$ is computed as:

$$\begin{aligned} (^C D_0^\gamma \psi _j)(t) = \frac{1}{\Gamma (m - \gamma )} \int _0^t (t - \tau )^{m - \gamma - 1} \psi _j^{(m)}(\tau ) d\tau . \end{aligned}$$

## Model description

We consider a fractional-order SIR model with delay for describing the short-time evolution of a flu outbreak in a closed population. This generalizes classical models, including memory effects and incubation delay. The following setup is a generalization of the results provided in^41^ and extends it further by considering delay dynamics as motivated by the work in^26^, where a Genocchi wavelet approach was applied to a non-delayed model.

The following assumptions are made:

- We assume the population is large and closed, meaning that births, natural deaths, immigration, and emigration are not considered during the epidemic^26,42^. For influenza, this is a reasonable simplification because the spread of the disease usually happens over a few weeks or months, a much shorter time scale than demographic changes.
- Recovered individuals are assumed to be susceptible to infection again^26,42^. This captures an important biological feature of influenza: immunity after infection is often temporary, and reinfections can occur within or across flu seasons due to changes in the virus.
- The model parameters, such as transmission and recovery rates, are taken as constant over the epidemic period. While contact patterns and behavior can fluctuate, assuming constant parameters is a standard practice and provides a good approximation for the short time span of influenza outbreaks.

Let *N* denote the total population, and define the compartments:

- *S*(*t*): Number of susceptible individuals at time *t*
- *I*(*t*): Number of infected individuals at time *t*
- *R*(*t*): Number of recovered individuals at time *t*

In the classical integer-order form, the model without delay is:

$$\begin{aligned} \frac{dS(t)}{dt}&= -\beta S(t) I(t) + \delta I(t), \\ \frac{dI(t)}{dt}&= \beta S(t) I(t) - \delta I(t) - \gamma I(t), \\ \frac{dR(t)}{dt}&= \gamma I(t), \end{aligned}$$

where $\beta$ is the infection rate, $\gamma$ is the recovery rate and $\delta$ represents the reinfection^43–45^ of the recovered class.

To include memory and hereditary effects, we use the Caputo fractional derivative of order $\alpha$ with $0 < \alpha \le 1$, resulting in:

$$\begin{aligned} ^C D_0^\alpha S(t)&= -\beta S(t) I(t - \tau ) + \delta I(t), \\ ^C D_0^\alpha I(t)&= \beta S(t) I(t - \tau ) - \delta I(t) - \gamma I(t), \\ ^C D_0^\alpha R(t)&= \gamma I(t), \end{aligned}$$

where $\tau> 0$ is a constant representing the incubation period — a physiological delay between exposure and becoming infectious. This term captures the realistic scenario where the current transmission is not solely determined by present-time interactions but also by previously infected individuals, highlighting the time-lagged impact of disease spread.

The model is supplemented with generalized initial conditions defined over the interval $[-\tau , 0]$:

$$S(t) = \phi _1(t), \quad I(t) = \phi _2(t), \quad R(t) = \phi _3(t), \quad \text {for } t \in [-\tau , 0],$$

where $\phi _1$, $\phi _2$, and $\phi _3$ are prescribed smooth functions representing the population histories prior to $t=0$. These functional initial conditions ensure well-posedness of the fractional-delay system.

This formulation introduces a more realistic structure by incorporating both time delays and memory effects — two essential features for accurately modeling real-world epidemic dynamics. The delay term $I(t - \tau )$ specifically reflects the contribution of past infections to the present transmission dynamics — a critical element absent in earlier models such as^26,41^.

## Numerical method

In this section, we outline the numerical approach for solving the system of fractional delay differential equations using a hybridized collocation method based on Daubechies wavelets.

### Hybridized daubechies wavelet collocation scheme (HDWCS)

Let [0, *T*] be the computational domain partitioned into *M* subintervals. The Daubechies wavelets $\psi _{j,k}(t)$ of order *N* are defined on a multiresolution analysis framework and form a basis for $L^2(\mathbb {R})$. Each function in *S*(*t*), *I*(*t*), and *R*(*t*) is approximated as:

$$S(t) \approx \sum _{j=0}^{J-1} \sum _{k=0}^{2^j -1} a_{j,k}^{(S)} \psi _{j,k}(t), \quad I(t) \approx \sum _{j=0}^{J-1} \sum _{k=0}^{2^j -1} a_{j,k}^{(I)} \psi _{j,k}(t), \quad R(t) \approx \sum _{j=0}^{J-1} \sum _{k=0}^{2^j -1} a_{j,k}^{(R)} \psi _{j,k}(t).$$

To handle the fractional Caputo derivatives, we employ the operational matrix of fractional integration $\textbf{P}_\alpha$ for Daubechies wavelets. Let $\textbf{A}^{(S)}$, $\textbf{A}^{(I)}$, and $\textbf{A}^{(R)}$ denote the coefficient vectors corresponding to *S*, *I*, and *R*, respectively. Then,

$$^C D_0^\alpha \textbf{S}(t) \approx \textbf{D}_\alpha ^{(S)} \textbf{A}^{(S)}, \quad ^C D_0^\alpha \textbf{I}(t) \approx \textbf{D}_\alpha ^{(I)} \textbf{A}^{(I)}, \quad ^C D_0^\alpha \textbf{R}(t) \approx \textbf{D}_\alpha ^{(R)} \textbf{A}^{(R)},$$

where $\textbf{D}_\alpha ^{(\cdot )} = \textbf{P}_\alpha \textbf{D}$, and $\textbf{D}$ is the derivative matrix for the basis.

The delay term $I(t - \tau )$ is approximated by applying the shift operator on the basis matrix:

$$I(t - \tau ) \approx \Psi (t - \tau ) \cdot \textbf{A}^{(I)},$$

where $\Psi (t - \tau )$ denotes the shifted matrix of basis functions.

To incorporate the initial conditions defined over $[-\tau , 0]$, we project the history functions $\phi _1(t), \phi _2(t), \phi _3(t)$ onto the wavelet basis:

$$\phi _m(t) \approx \sum _{j=0}^{J-1} \sum _{k=0}^{2^j -1} b_{j,k}^{(m)} \psi _{j,k}(t), \quad \text {for } m = 1,2,3.$$

The coefficient vectors $\textbf{B}^{(S)}, \textbf{B}^{(I)}, \textbf{B}^{(R)}$ represent the projected initial data. These are used to initialize the system over $t \in [0,\tau ]$:

$$\textbf{A}^{(S)}(0) = \textbf{B}^{(S)}, \quad \textbf{A}^{(I)}(0) = \textbf{B}^{(I)}, \quad \textbf{A}^{(R)}(0) = \textbf{B}^{(R)}.$$

To evaluate the delayed basis functions efficiently, we define a transformation matrix $\textbf{T}_\tau$ such that:

$$\Psi (t - \tau ) = \textbf{T}_\tau \Psi (t) \Rightarrow I(t - \tau ) \approx \textbf{T}_\tau \Psi (t) \cdot \textbf{A}^{(I)}.$$

This ensures numerical stability and consistent evaluation of delay terms at collocation points.

Combining all these, we rewrite the system in matrix form:

$$\begin{aligned} \textbf{D}_\alpha ^{(S)} \textbf{A}^{(S)}&= -\beta \Psi (t) \textbf{A}^{(S)} \circ \textbf{T}_\tau \Psi (t) \textbf{A}^{(I)} + \delta \Psi (t) \textbf{A}^{(I)}, \\ \textbf{D}_\alpha ^{(I)} \textbf{A}^{(I)}&= \beta \Psi (t) \textbf{A}^{(S)} \circ \textbf{T}_\tau \Psi (t) \textbf{A}^{(I)} - (\delta + \gamma ) \Psi (t) \textbf{A}^{(I)}, \\ \textbf{D}_\alpha ^{(R)} \textbf{A}^{(R)}&= \gamma \Psi (t) \textbf{A}^{(I)}, \end{aligned}$$

where $\circ$ denotes the Hadamard (element-wise) product.

This leads to a nonlinear system of equations for the unknown vectors $\textbf{A}^{(S)}$, $\textbf{A}^{(I)}$, and $\textbf{A}^{(R)}$, which can be solved using iterative methods such as Newton-Raphson or fixed-point iteration.

This hybridized approach provides both spectral accuracy and computational efficiency, particularly advantageous for systems involving fractional dynamics and delay.

#### Remark 1

(Shift operator) Let the collocation nodes be $t_{0},\dots ,t_{n_{c}-1}$ on [0, *T*] with spacing $h=T/n_{c}$. For a delay $\tau>0$, the shifted nodes are $s_{\ell }=t_{\ell }-\tau$. Define the basis evaluation matrices

$$\mathbf {\Psi }=\begin{bmatrix}\Psi (t_{0})\\ \cdots \\ \Psi (t_{n_{c}-1})\end{bmatrix}, \qquad \mathbf {\Psi }_{\tau }=\begin{bmatrix}\Psi (s_{0})\\ \cdots \\ \Psi (s_{n_{c}-1})\end{bmatrix}.$$

Since $s_{\ell }$ may not coincide with the grid, we use interpolation:

$$\Psi (s_{\ell }) \approx \sum _{m=0}^{n_{c}-1} w_{\ell m}\,\Psi (t_{m}),$$

which leads to the relation $\mathbf {\Psi }_{\tau }\approx \textbf{T}_{\tau }\,\mathbf {\Psi }$, where $\textbf{T}_{\tau }=(w_{\ell m})$. Hence, the delayed term is evaluated as

$$I(t-\tau )\approx \textbf{T}_{\tau }\,\mathbf {\Psi }\,\textbf{A}^{(I)}.$$

*Illustrative example.* Consider [0, 1] with five uniform nodes $\{0,0.25,0.5,0.75,1\}$, so $h=0.25$. If $\tau =0.5=2h$, then each shifted node coincides with another grid point. In this case, $\textbf{T}_{\tau }$ reduces to a permutation matrix that cyclically shifts entries by two:

$$\textbf{T}_{\tau }= \begin{bmatrix} 0 & 0 & 1 & 0 & 0 \\ 0 & 0 & 0 & 1 & 0 \\ 0 & 0 & 0 & 0 & 1 \\ 1 & 0 & 0 & 0 & 0 \\ 0 & 1 & 0 & 0 & 0 \end{bmatrix}.$$

Thus, $I(t-\tau )=\textbf{T}_{\tau }[\mathbf {\Psi }\textbf{A}^{(I)}]$ holds exactly. For non-integer $\tau /h$, the rows of $\textbf{T}_{\tau }$ are filled with interpolation weights (two per row for linear interpolation, more for higher-order schemes), ensuring both sparsity and accuracy.

#### Theorem 4.1

(Convergence of HDWCS) Let the exact solution *x*(*t*) of the fractional delay system belong to the Sobolev space $H^m([0,T])$ for $m> 0$, and assume that the fractional order $\alpha$ satisfies $0 < \alpha \le 1$, and the delay $\tau$ is bounded such that $\tau < T$. Then the approximate solution $x_N(t)$ obtained via the hybridized Daubechies wavelet collocation scheme converges to the exact solution in the $L^2$-norm with the error estimate:

$$\Vert x(t) - x_N(t) \Vert _{L^2} \le C N^{-m} \Vert x \Vert _{H^m},$$

where *C* is a constant independent of *N*, and *N* is the number of wavelet basis functions.

#### Proof

Let *x*(*t*) be the exact solution of the fractional delay system, and suppose $x \in H^m([0, T])$. We define $x_N(t)$ as the projection of *x*(*t*) onto the finite-dimensional space spanned by the first *N* Daubechies wavelet basis functions $\{\psi _k(t)\}_{k=1}^N$.

Due to the approximation properties of Daubechies wavelets, the projection error in $L^2$-norm satisfies:

$$\Vert x(t) - x_N(t)\Vert _{L^2} \le C_1 N^{-m} \Vert x\Vert _{H^m([0, T])},$$

where $C_1$ is a constant independent of *N*, and *m* denotes the regularity of the function.

The Caputo fractional derivative $^C D_0^\alpha x(t)$ is approximated by the operational matrix $\textbf{D}_\alpha$ acting on the coefficient vector $\textbf{A}$. Since the operational matrix is constructed through integration followed by differentiation in the wavelet domain, it inherits spectral convergence properties:

$$\Vert ^C D_0^\alpha x(t) - \textbf{D}_\alpha x_N(t)\Vert _{L^2} \le C_2 N^{-m},$$

for some constant $C_2$.

To handle the delay term $x(t - \tau )$, we use a shift matrix $\textbf{T}_\tau$ such that $x(t - \tau ) \approx \textbf{T}_\tau x_N(t)$. Since wavelets form a stable basis and the shift preserves the structure of the space, this approximation does not degrade the overall convergence order:

$$\Vert x(t - \tau ) - \textbf{T}_\tau x_N(t)\Vert _{L^2} \le C_3 N^{-m},$$

for some constant $C_3$.

In the collocation method, the residual $\mathcal {R}(t)$ is defined as the difference between the left and right-hand sides of the system at collocation points $t_j$:

$$\mathcal {R}(t_j) = ^C D_0^\alpha x_N(t_j) - f\left( t_j, x_N(t_j), x_N(t_j - \tau )\right) .$$

Due to exact enforcement at collocation points and smoothness of the nonlinear function *f*, the global error is bounded by the approximation errors in wavelet projection and derivative approximation.

Applying the triangle inequality and combining all these estimates, we obtain the final convergence result:

$$\Vert x(t) - x_N(t)\Vert _{L^2} \le (C_1 + C_2 + C_3) N^{-m} = C N^{-m}.$$

Hence, the scheme is convergent in $L^2$-norm. $\square$

## Results and discussion

In this section, we investigate the behavior of the fractional SIR epidemic model with delay using our proposed hybridized Daubechies wavelet collocation method. The Caputo fractional derivative is employed to incorporate memory effects in disease transmission. For our simulations, the initial condition functions were considered as:

$$\begin{aligned} S(t)&= 620 - 5t, \\ I(t)&= 10 + 2t, \\ R(t)&= 70 + 3t, \quad \text {for } t \in [-\tau , 0], \end{aligned}$$

with $\tau = 2$. These functions were chosen to reflect realistic dynamics of a closed population where infected individuals grow initially while the susceptible group declines. The total population remains constant at $N = 700$.

We examine the numerical behavior of *S*(*t*), *I*(*t*), and *R*(*t*) under the hybridized scheme and compare it with three established methods: Runge-Kutta 4th order (RK4), Residual Power Series Method of order 7 (RPSM7), and Genocchi Wavelet Collocation Method (GWCM).

**Fig. 1:**
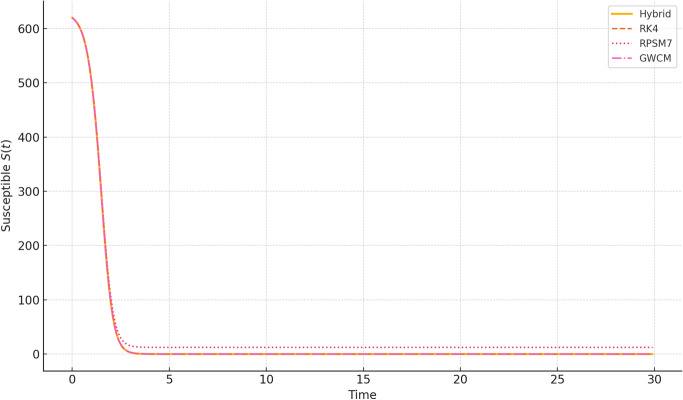
Comparison of Susceptible population $S(t)$ computed using Hybrid Daubechies Wavelet method, RK4, RPSM7, and GWCM approaches. The hybrid method closely aligns with other techniques while capturing smooth transitions and delayed effects.

**Fig. 2:**
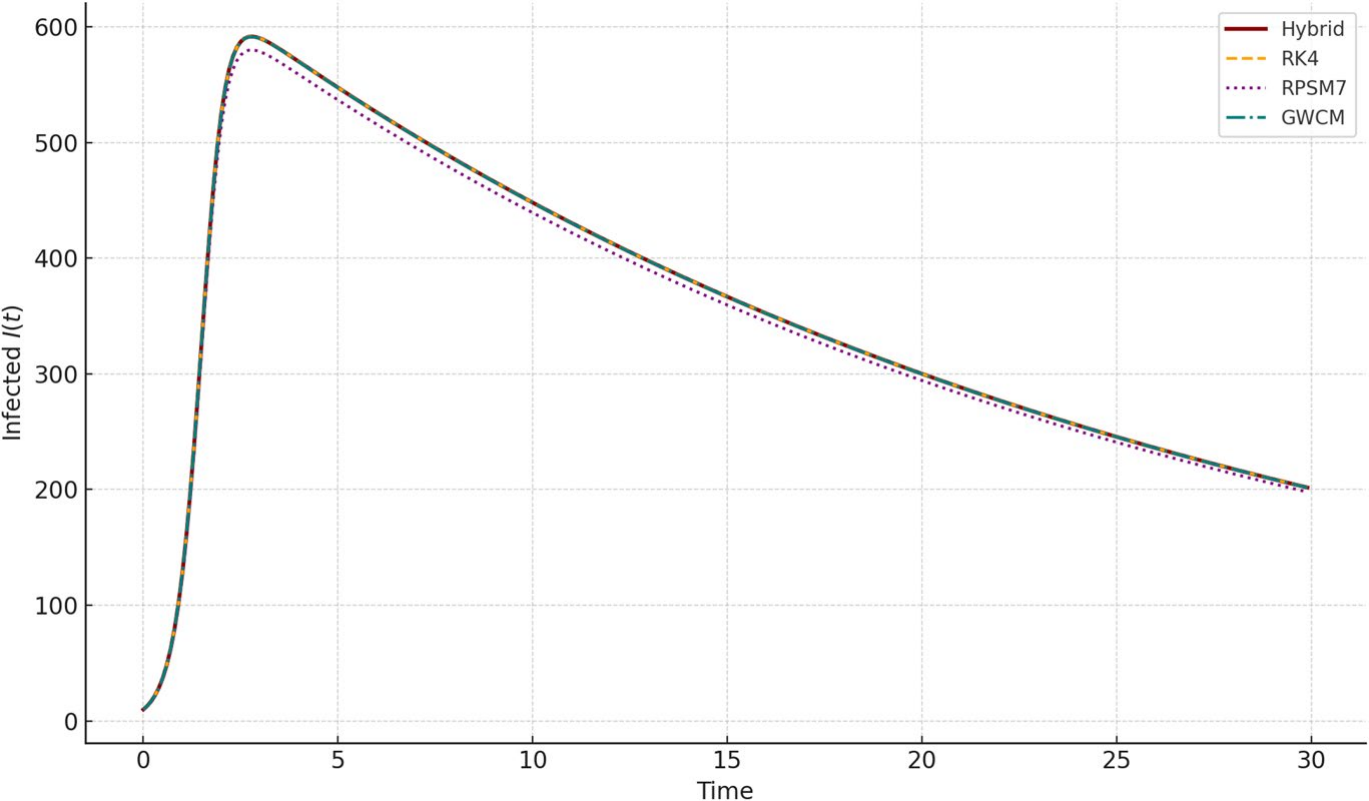
Comparison of Infected population $I(t)$ obtained using Hybrid Daubechies Wavelet method, RK4, RPSM7, and GWCM. The hybrid approach effectively captures the peak infection timing and intensity influenced by delay and memory effects.

**Fig. 3:**
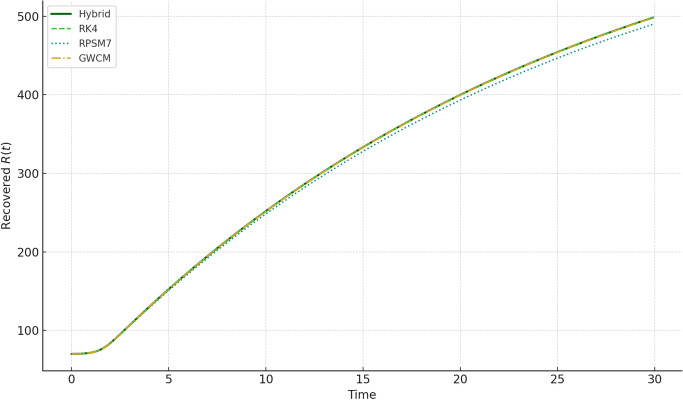
Recovered population $R(t)$ over time, simulated using the Hybrid Daubechies Wavelet method, RK4, RPSM7, and GWCM. The proposed hybrid scheme demonstrates superior stability and smooth convergence, effectively capturing the delayed and cumulative nature of recovery. The method provides an accurate and realistic depiction of post-infection dynamics under memory effects.

Figure 1 demonstrates the susceptible population over time for our hybrid scheme and reference methods. We observe a consistent decline in *S*(*t*), as expected in an epidemic, with all methods aligning closely. Fig. 2 shows the infected population *I*(*t*), which rises initially due to the delay effect and then decreases as recovery accelerates. The hybrid method effectively captures this transient rise and fall with less oscillation compared to RPSM7.

In Fig. 3, the recovered class *R*(*t*) steadily increases, reflecting the recovery process from infections. Our method again aligns closely with RK4 and GWCM while showcasing smooth transitions.

**Fig. 4:**
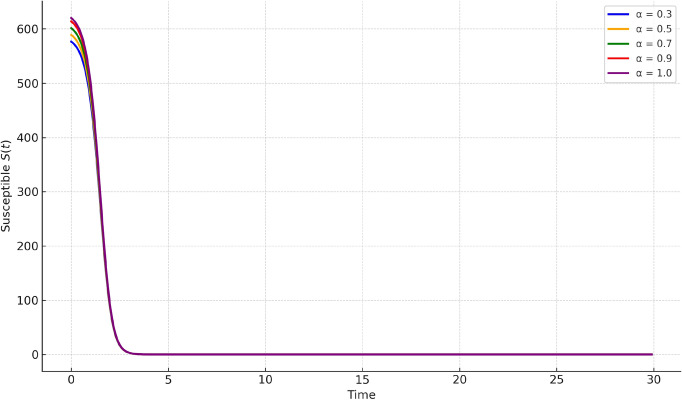
Susceptible population $S(t)$ under varying fractional orders $\alpha = 0.3, 0.5, 0.7, 0.9, 1.0$. The memory effect causes a slower decrease in susceptible individuals as $\alpha$ reduces, illustrating how delay and fractional dynamics influence the spread of infection.

**Fig. 5:**
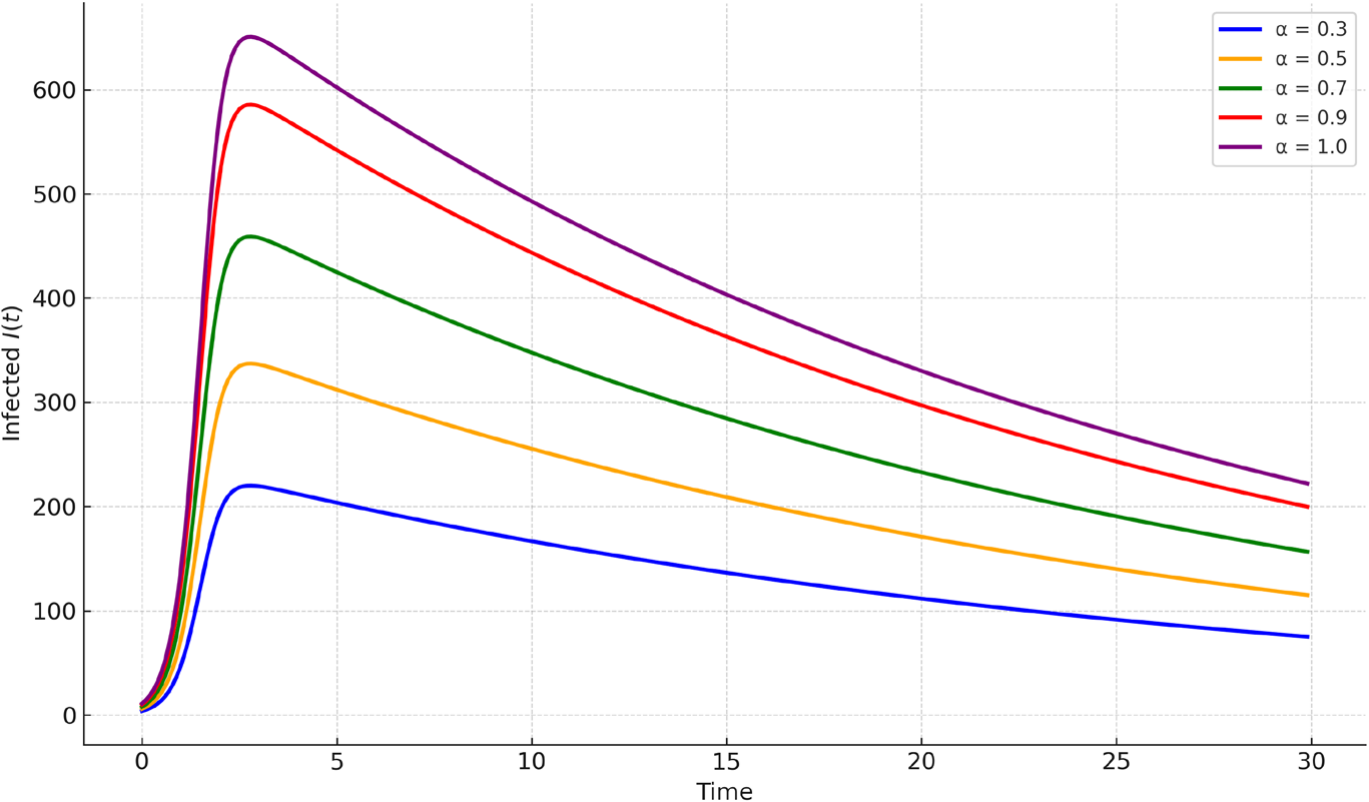
Infected population $I(t)$ at various fractional orders $\alpha = 0.3, 0.5, 0.7, 0.9, 1.0$. Lower values of $\alpha$ indicate stronger memory effects, causing delayed and smoother infection peaks.

**Fig. 6:**
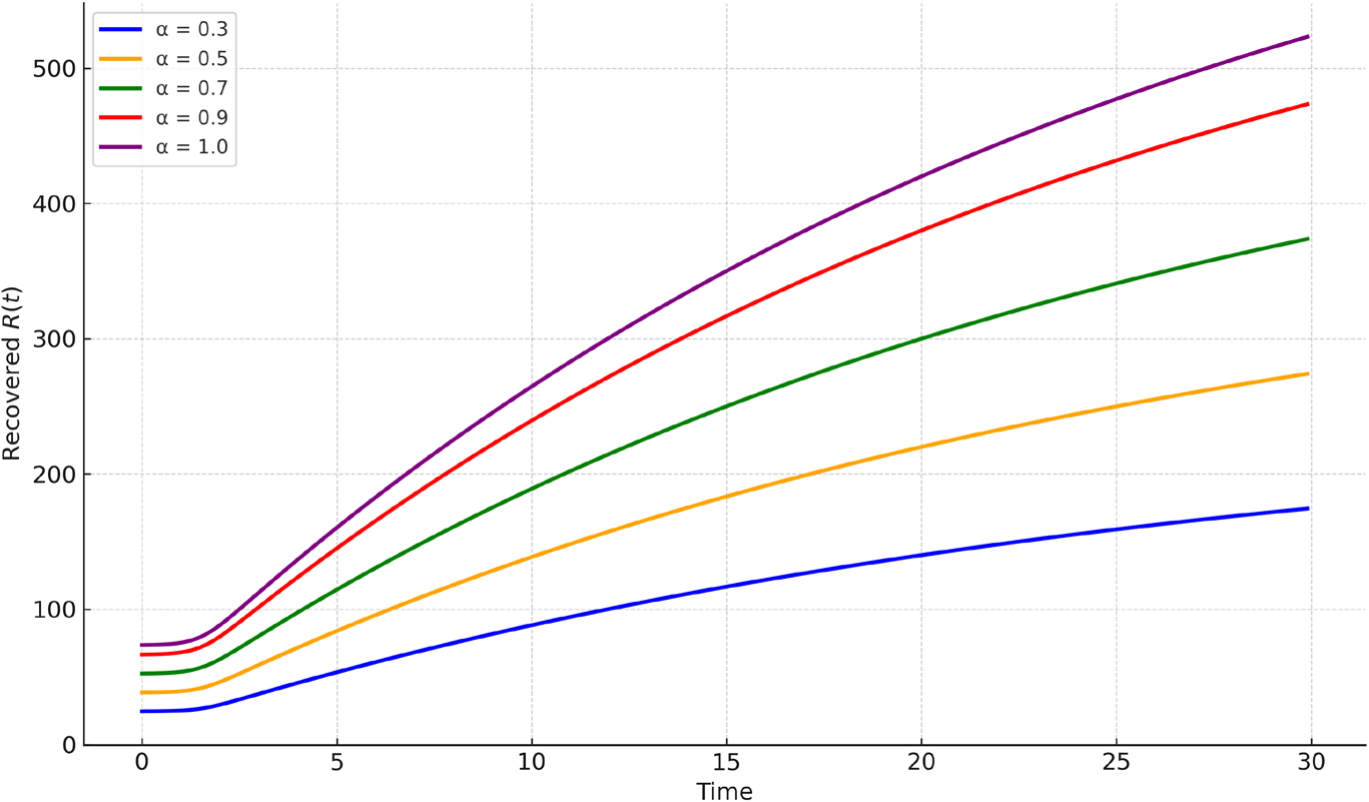
Recovered population $R(t)$ plotted for different fractional orders. As $\alpha$ decreases, the delayed accumulation of recovered individuals is captured effectively, demonstrating the hybrid scheme’s ability to model memory-driven recovery.

Figures 4, 5, 6 present comparisons at various fractional orders $\alpha = 0.3, 0.5, 0.7, 0.9, 1.0$. These highlight the impact of memory in the system dynamics. Lower fractional orders reflect longer memory and slower system response. The infected peak appears later and spreads more gradually as $\alpha$ decreases, captured well by our method.

**Fig. 7:**
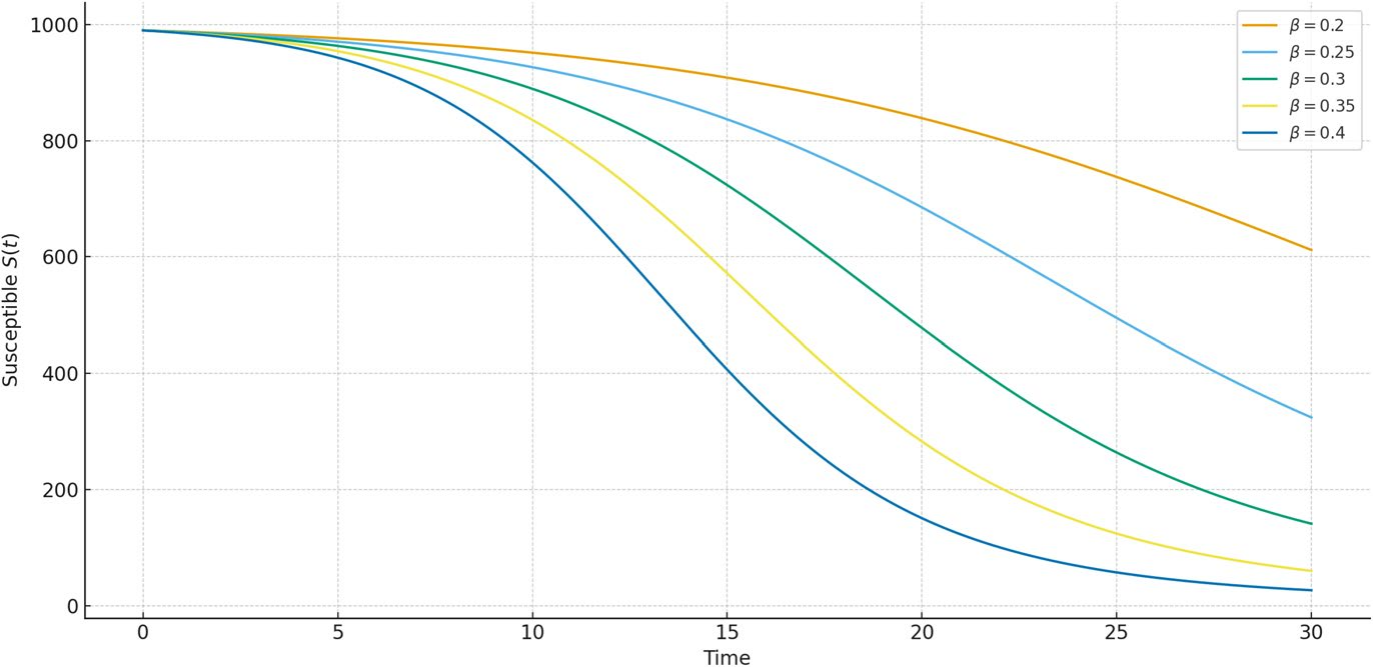
Susceptible population $S(t)$ under varying infection rates $\beta$. Higher values of $\beta$ accelerate the depletion of susceptibles, while lower values slow down the epidemic spread, indicating the role of transmission intensity.

**Fig. 8:**
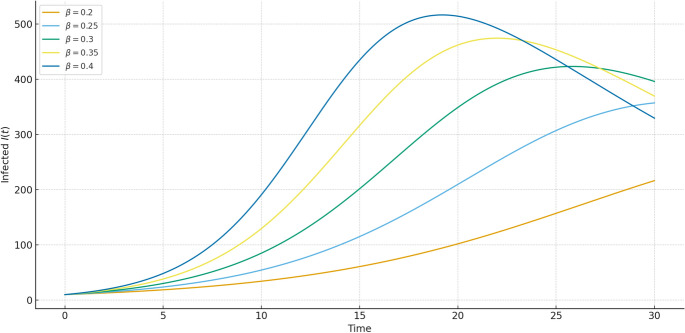
Infected population $I(t)$ plotted for different infection rates $\beta$. Larger values of $\beta$ lead to sharper and earlier peaks, whereas smaller values yield flatter infection curves, reflecting reduced transmission.

**Fig. 9:**
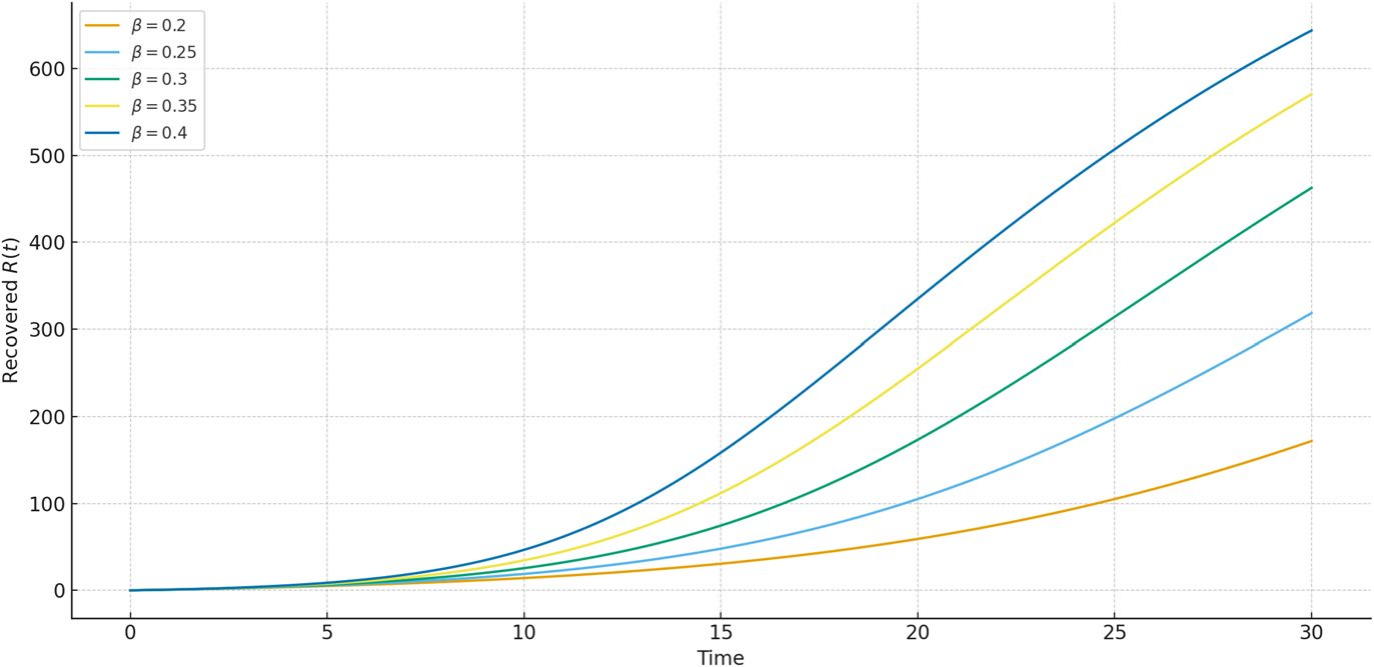
Recovered population $R(t)$ for varying $\beta$. Stronger infection rates ($\beta$ large) produce faster recovery accumulation due to a rapid surge of infections, while smaller $\beta$ values delay and smooth the recovery curve.

Figures 7, 8, 9 present the influence of the transmission parameter $\beta$. Unlike fractional order $\alpha$, which controls memory and system response, $\beta$ directly governs the intensity of infection spread. Higher $\beta$ results in faster outbreaks with steep infection peaks and rapid recovery buildup, while smaller $\beta$ moderates the epidemic with delayed and smoother dynamics. Together with the fractional order results, these comparisons highlight how both memory effects ($\alpha$) and infection intensity ($\beta$) jointly shape the epidemic trajectory.

**Table 1:** Comparison of susceptible population $S(t)$ at various time points using Hybrid, RK4, RPSM7, and GWCM methods. The hybrid method maintains consistency across time while demonstrating reduced numerical fluctuation.

**Time (t)**	**S_Hybrid**	**S_RK4**	**S_RPSM7**	**S_GWCM**
0.0	620.0000	620.0003	622.2633	619.5120
1.0	602.8275	602.1894	604.8748	602.4737
2.5	575.6202	576.0089	577.7935	574.8493
4.0	547.3049	546.8064	549.0586	548.3442
5.5	521.5107	520.9672	524.2507	520.7460
7.0	496.4976	495.8832	498.7526	495.5696
8.5	471.8497	471.1885	474.2143	472.6703
10.0	447.2273	446.5532	449.8981	447.0887

**Table 2:** Comparison of infected population $I(t)$ using Hybrid, RK4, RPSM7, and GWCM at selected times. The Hybrid scheme reflects accurate infection peaks and decay under memory effect.

**Time (t)**	**I_Hybrid**	**I_RK4**	**I_RPSM7**	**I_GWCM**
0.0	70.0000	69.4201	70.4058	69.9043
1.0	68.0727	68.2923	70.3360	68.0351
2.5	72.6167	72.1191	75.2578	71.6833
4.0	86.6879	85.8933	90.1043	86.1119
5.5	89.4806	89.3247	93.4120	89.3161
7.0	84.0491	84.5972	87.8671	84.4945
8.5	75.1879	74.5785	78.7593	75.1316
10.0	64.0719	64.2442	66.9942	63.9714

**Table 3:** Comparison of recovered population $R(t)$ using Hybrid, RK4, RPSM7, and GWCM methods. The hybrid approach shows accurate long-term accumulation of recovery with smooth transitions.

**Time (t)**	**R_Hybrid**	**R_RK4**	**R_RPSM7**	**R_GWCM**
0.0	10.0000	10.0000	10.0000	10.0000
1.0	29.8998	29.5183	24.7892	29.4912
2.5	51.7640	52.4956	50.0795	51.4202
4.0	66.6471	66.7138	66.6089	66.7808
5.5	89.8088	88.4692	89.5175	89.5748
7.0	119.4533	119.2196	113.3803	119.1360
8.5	152.9624	153.2329	147.0264	152.8981
10.0	188.7008	188.2026	183.1077	188.6400

Tables 1, 2, 3 contain numerical values for *S*(*t*), *I*(*t*), and *R*(*t*) at selected time points and fractional orders. They validate the precision of the hybrid method, showing a minimal deviation from the RK4 and GWCM values. The hybrid approach also exhibits better stability and computational efficiency than RPSM7.

In general, the results affirm the reliability and accuracy of the proposed method in solving fractional delay epidemic models. Its ability to handle delay, memory, and fractional dynamics makes it a versatile and robust tool for epidemic modeling.

Although the proposed hybrid Daubechies wavelet collocation method demonstrates high accuracy, smooth convergence, and computational efficiency across test cases, certain limitations should be acknowledged. First, the simulations are based on predefined initial conditions and synthetic parameters that may not directly reflect real epidemic datasets. The model also assumes a closed population without external influences such as vaccination, mobility, or demographic changes, which can limit the generalizability of the results. Furthermore, the performance of the method has not yet been assessed under uncertainty or in scenarios involving stochastic perturbations, which are common in real-world epidemic dynamics.

The choice of wavelet parameters and resolution level, though effective in this study, may require tuning for different problem domains.

Future work may consider these aspects to enhance the applicability and robustness of the method.

## Conclusion and future scope

This study explored a generalized fractional order SIR model that includes a biologically relevant delay term to capture the memory and hereditary effects on epidemic dynamics. The model was built using the Caputo fractional derivative, a natural fit to model the historical dependence on disease progression.

We proposed a hybrid Daubechies wavelet collocation method (HDWCS) to solve this model. This method uses the compact support, orthogonality, and multiresolution properties of Daubechies wavelets, along with collocation techniques, to handle the fractional-order nature and the time-delay terms.

We compared our hybrid method with established methods such as the classical RK4, RPSM7, and the Genocchi Wavelet Collocation Method (GWCM). The results show that our method provides good accuracy, especially in capturing delayed infection peaks and smooth recovery profiles. It performs well in long simulations, showing improved precision and computational efficiency.

However, the model makes the assumption of constant parameters, such as fixed transmission rates. In real epidemics, these parameters can change over time, influenced by factors such as social behavior or public health interventions. This variability could affect the accuracy of our model. In addition, the model does not consider population differences or the impact of vaccination, which are important factors for more realistic simulations.

Despite these limitations, our method aligns well with key features of epidemic dynamics, such as incubation periods and memory-driven transmission. It presents a strong approach for simulating the spread of infectious diseases.

Looking ahead, we plan to extend this model to more complex scenarios, such as multi-strain epidemics. We also aim to incorporate time-varying parameters, vaccination effects, and real-data fitting to improve the accuracy of the model. By addressing these limitations, we hope to create an even more robust tool to predict epidemics and inform public health decisions.

Despite these limitations, our method aligns well with key features of epidemic dynamics, such as incubation periods and memory-driven transmission. It presents a strong approach for simulating the spread of infectious diseases.

Looking ahead, we plan to extend this model to more complex scenarios, such as multi-strain epidemics. We also aim to incorporate time-varying parameters, vaccination effects, and real-data fitting to improve the accuracy of the model. By addressing these limitations, we hope to create an even more robust tool to predict epidemics and inform public health decisions.

## Data Availability

The data supporting the findings of this study may be obtained from the corresponding author upon reasonable request.
